# Differences in Dietary Patterns of Adolescent Patients with IBD

**DOI:** 10.3390/nu13093119

**Published:** 2021-09-06

**Authors:** Justyna Kikut, Karolina Skonieczna-Żydecka, Diana Sochaczewska, Agnieszka Kordek, Małgorzata Szczuko

**Affiliations:** 1 Department of Human Nutrition and Metabolomics, Pomeranian Medical University in Szczecin, Broniewskiego 24 Street, 71-460 Szczecin, Poland; justyna.kikut@pum.edu.pl; 2 Department of Biochemical Sciences, Pomeranian Medical University in Szczecin, Broniewskiego 24 Street, 71-460 Szczecin, Poland; karzyd@pum.edu.pl; 3 Department of Neonatology, Pomeranian Medical University in Szczecin, 72-010 Police, Poland; Diana.sochaczewska@pum.edu.pl (D.S.); agkordek@pum.edu.pl (A.K.)

**Keywords:** inflammatory bowel disease, Crohn’s disease, ulcerative colitis, diet, nutrition, teenagers, adolescents

## Abstract

Inflammatory bowel disease (IBD) includes Crohn’s disease (CD) and ulcerative colitis (UC). The prevalence of both in pediatric populations has been constantly increasing. This study aimed to analyze the diet of adolescent patients with IBD in comparison to healthy controls and the current dietary standards for the Polish population to further their optimal supplementation regimen. The study group consisted of 53 patients (21 girls and 32 boys) with IBD (CD: n = 27; UC: n = 26) at a mean age of 15.4 ± 2.4 and 14.7 ± 2.2, years for girls and boys, respectively. The control group (CG) consisted of 20 patients, and 72 h of recall diaries on nutrition were collected. The nutritional data were analyzed in the Dieta 6D dietary program. When compared to Polish dietary standards, the largest differences girls with IBD and boys with IBD were found for the intake of energy (61.9 and 71.9%), iodine (61.9 and 62.6%), folates (76.2 and 87.5%), vitamin D (100 and 96.9%), potassium (61.9 and 59.4%), and calcium (85.7 and 93.8%). The overconsumption of saturated fatty acids (SFA) (61.9 and 56.3%) and sodium (76.2 and 90.6%) in girls and boys, respectively, was noted. In relation to girls with CG, girls with IBD showed a significantly higher intake of energy (1751. 3 vs. 1558.6 *p* = 0.0224), total protein (71.3 vs. 56.2 *p* = 0.0217), animal protein (47.8 vs. 34.5 *p* = 0.0183), total carbohydrates (237.3 vs. 196.1 *p* = 0.0442), and assimilable carbohydrates (219.8 vs. 180.5 *p* = 0.7921). Boys in the CG consumed significantly more calcium (851.8 vs. 432 *p* = 0.0006), phosphorus (1024.3 vs. 1357.5 *p* = 0.0431), lactose (11.6 vs. 6.1 *p* = 0.0016), and riboflavin (1.7 vs. 1.3 *p* = 0.0123) compared to boys with IBD. Dietician care should therefore be mandatorily provided alongside outpatient care. Based on our results, we suggest that supplementation with the selected components be considered.

## 1. Introduction 

Inflammatory bowel disease (IBD) includes Crohn’s disease (CD) and Ulcerative Colitis (UC). The disease is characterized by chronic non-infectious inflammation of the gastrointestinal tract (GT) [1]. UC mainly affects the large intestine, whereas CD may involve the entire gastrointestinal tract [2]. UC is defined as an idiopathic, chronic, and recurrent inflammatory disease of the colon. Inflammation of the mucosa in UC extends proximally along the entire colon to include the rectum. The degree of mucosal involvement here determines the course of the disease. Dysregulated immune response of the intestinal mucosa, influenced by the interaction of genetic and environmental factors, is thought to be a predictor of UC [3,4].

Currently, a relevant increase in the prevalence of IBD in children has been noted [5]. The group under 18 years of age represents an estimated 10–15% of diagnosed patients [2]. A particularly higher incidence rate has been observed in developed countries. Interestingly, a similarly higher incidence rate has been observed in children who emigrate from developing countries compared to minors from developed countries [6]. This is related, among other things, to Western diets being characterized by excessive consumption of meat and fat and an insufficient intake of fiber, fruit, and vegetables [7,8]. Excessive intake of trans fatty acids may be associated with systemic inflammation and risk of UC [9]. CD showed an association between excessive intake of total fat, animal-derived fat, and n-6, with a higher incidence of disease [10]. 

The pathophysiology of IBD is still not completely understood. It has been suggested that the disease occurs in genetically susceptible individuals as a result of an uncontrolled immune system response to a trigger factor [1]. Other factors include environmental factors such as diet or antibiotic use and immunological factors, all of which have been linked to microbiome structure and function disruptions [11,12]. These factors outweigh the genetic ones because genetics alone cannot explain such a significant increase in IBD incidence [2,5]. 

According to the recommendations of the European Society for Clinical Nutrition and Metabolism (ESPEN), nutritional care is a key component of therapy for patients with IBD. CD patients especially are at a higher risk of developing malnutrition due to the possible involvement of each gastrointestinal tract segment. Moreover, there are no specific nutritional guidelines or recommendations regarding nutrition for individuals with IBD. The diet is usually based on the patient’s individual condition [13].

With chronic inflammation and impaired absorption, the risk of deficiencies in micro and macro dietary components increases. Malabsorption is correlated with mucosal function abnormalities such as impaired transport across the epithelium and reduced intestinal barrier integrity [14]. IBD patients make use of a variety of dietary elimination strategies. Up to 70% of patients continue with elimination diets after remission for fear of re-exacerbating the disease [15,16]. Moreover, their relationship with food, i.e., behaviors or beliefs about food, has a major impact on their social life [15,16]. On the other hand, other studies show that 57% of the respondents indicate diet as a predictor of relapse and even 66% of the patients introduce various types of restrictions in the consumption of favorite products [17]. Similarly, in the Crooks et al. study, 59% of UC patients shunned their favorite foods for fear of exacerbating their disease, and 23% of patients followed lactose- and gluten-free diets [18]. Unfortunately, the disadvantage of elimination diets and highly restrictive diets is that they significantly increase the possibility of deficiencies in micro- and macro-components of the diet. Additionally, the variety of proposed dietary recommendations often misleads patients by increasing the use of unnecessary eliminations [19]. Patients with IBD also have food intolerances, predominantly to gluten, lactose, or fructose, which increases the popularity of elimination diets [20,21]. 

Proper nutrition is particularly important for the proper development of youth. Identifying inappropriate eating habits at an early age can reduce the risk of their repetition into adulthood. An adequate dietary intake of nutrients supports the healing process and an inadequately managed diet can become highly deficient and lead to impaired physical, intellectual, and mental development. In addition, as mentioned above, abnormal attitudes toward eating can negatively impact social competency. 

Therefore, the aim of this study was to assess the dietary intake of energy, macro-, and micronutrients by adolescents with IBD compared to Polish reference standards and control group (CG) intake. The study also aimed to identify possible nutritional deficiencies in patients with IBD and to identify components for which supplementation should be considered [22].

A pilot study of ours on IBD and celiac disease has found no significant differences in the nutritional habits between CD and UC. In contrast, differences in nutrition intake between IBD and celiac disease have been shown [23]. Thus, this article analyzes the diets of adolescents with IBD in terms of gender.

## 2. Material and Methods

### 2.1. Study Group (IBD Group)

The study presents a single-center analysis of the diets of adolescent patients with IBD. The study group included adolescent patients with a mean age of 15.4 ± 2.4 years for girls and 14.7 ± 2.2 for boys, hospitalized at the Department of Pediatrics, Haemato-Oncology and Pediatric Gastroenterology of the Pomeranian Medical University in Szczecin. Patient recruitment took place from January 2019 to January 2021. The study was approved by the Bioethics Committee of the Pomeranian Medical University (regulation no. KB-0012/131/18).

The study group consisted of 73 patients, while 20 patients refused to take part in the study. The study group consisted of 53 patients with IBD, including 27 patients with CD (8 girls and 19 boys) and 26 patients with UC (13 girls and 13 boys). The mean height and body weight were respectively 1.6 ± 0.1 and 54.5 ± 13.9 kg and for girls and 1.6 ± 0.2 and 52.7 ± 17.7 for boys. The differences in parameters were not statistically significant (*p*-values were, respectively 0.929, 0.709). The diagnosis of IBD was made based on endoscopic examination (using an Olympus camera) with histopathological evaluation of the intestinal tissue collected during endoscopic examination, which was one of the inclusion criteria for the study. In addition, patients had an ultrasound examination. Whereas in the case of Crohn’s disease, enteroclysis or enterography with CT imaging was additionally performed. Patients aged 10 to 18 years with mild to moderate IBD on standard oral nutrition and not specialized elimination diets (e.g., ModuLife, SCD, low FODMAP, vegan, vegetarian) were included in the study. Only patients with lactose intolerance who consumed lactose-free dairy products were included in the study (n = 10). Lactose intake was determined by interview and food diaries. Patients reported consuming dairy products without lactose. They did not eliminate dairy from their diet due to lactose intolerance. Exclusion criteria included the use of specialized elimination diets, other food allergies, and age below ten years.

The mean disease duration was 2.5 years in boys and 2.9 years in girls. In the UC and CD group, 19 and 20 patients, respectively, were in remission, and 7 patients were facing a period of disease exacerbation. The degree of disease was determined by a gastroenterologist on the basis of the Pediatric Crohn’s Disease Activity Index (PCDAI) Pediatric Ulcerative Colitis Activity Index (PUCAI) questionnaire. Remission of the disease was established by the attending physician through biochemical parameters of inflammation (erythrocyte sedimentation rate (ESR), C-reactive protein (CRP), fecal calprotectin). Patients were taking the following groups of drugs: corticosteroids (n = 12), immunosuppressants (n = 25), aminosalicylic acid medications (n = 42), and biological treatment (n = 2). Most patients maintained the habits acquired during disease exacerbations even after going into remission; therefore, their diets did not differ between periods. Among girls, 47.6% took vitamin D supplements (including 50% with CD and 46.2% with UC), 9.5% supplemented with a multivitamin complex (including 12.5% with CD and 7.7% with UC). Referring to minerals, 23.8% supplemented calcium (including 50% with CD and 7.7% with UC) and 9.5% of girls supplemented potassium. Among girls, only 15.4% of UC girls were taking iron supplements. In turn, in the boys’ group, vitamin D supplementation was taken by 75% (including 78.9% with CD and 69.2% with UC), vitamin complex by 9.4% (10.5% with CD and 7.7% with UC). Referring to minerals, calcium was supplemented by 25% of boys (21.1% from CD and 30.8% from UC), potassium by 15.6% (21.1% from CD and 7.7% from UC), iron by 6.3% (5.3% from CD, 7.7% from UC). Magnesium was supplemented by only 10.5% of boys with CD. Supplementation is not included in the daily intake.

### 2.2. Control Group (CG)

The control group was recruited randomly from age- and gender-matched healthy children from the local community in the winter of 2020/2021. General information about the study and its objectives was advertised via social media and paper flyers. Parent’s communicated their children’s willingness to participate by phone. During the telephone calls, it was confirmed that participating children met the following criteria: no changes in dietary habits in the three months prior to the study, including use of elimination diets; no chronic diseases, especially concerning the gastrointestinal tract; and no food intolerances or allergies. Overall, 32 parents gave consent for their children to take part in the study, but at last 20 children came for a face-to-face interview. The control group consisted of 10 girls and 10 boys, aged 14.3 ± 2.7 and 14 ± 3.2 years, respectively. The mean height and body weight were, respectively, 1.6 ± 0.1 and 51 ± 10.3 kg for girls, and 1.7 ± 0.1 and 52 ± 12.3 for boys. The differences in these parameters were not statistically significant (*p*-values were 0.342, 0.614, respectively).

Anthropometric measurements such as body weight (±0.1 kg) and height (±0.5 cm) were used to assess differences in the nutritional status of patients. A medical scale (Radwag WPT 60/150 OW, Poland) with a height gauge was used.

### 2.3. Diet Content Analysis

Dietary patterns were assessed based on recalled nutritional diaries of the 72 h prior to the assessment. The dietary interview was conducted by a qualified nutritionist. An album of products and meal photos was used to ensure the collection of reliable information regarding the size of the consumed meals [24]. Attempts were made to rule out recall error by using an album of products and meals photos and the record of each day’s food diary was verified by a dietitian in the presence of the patient/guardian. A total of 219 children’s nutritional diaries were analyzed, of which 159 were from children with IBD and 60 were from healthy children. The Dieta 6D software, recommended by the National Centre of Nutritional Education (Warsaw, Poland), was used for quantitative analysis. The program includes 58 micro- and macro-nutrients, including carbohydrates, fats and fatty acids, and animal and vegetable proteins, with 18 amino acids, 10 minerals, and 10 vitamins, as well as dietary energy, fiber, and cholesterol. The obtained data were compared with the current nutrition standards for the Polish population and with the control group [25]. 

### 2.4. The Comparison with Current Nutrition Standards 

The results obtained were compared with the current nutrition standards for the Polish population. The norms were used according to the average age for both genders, respectively. The results were related to the estimated average requirement (EAR), or if this was not established to the adequate intake (AI).The reference of intake levels to norms is presented with a ±10% margin. The mean age of 13–15 years for girls and boys, respectively, was taken as the reference point for nutritional standards. To compare energy intake to norms, the average body mass and average age of the study group were used for boys and girls, respectively. All patients in the study group had low physical activity limited to a maximum of 2–4 h per week, which was also contributed to by the current epidemiological situation. The average activity of the group was set at a physical activity level of 1.50 for girls and 1.55 for boys. 

### 2.5. Statistical Analysis

Statistical analysis was performed using Statistica 13.3 software (Statsoft, Krakow, Poland). The distribution of the continuous data was tested using the Shapiro–Wilk test. Since most data in the study group did not differ significantly from the normal distribution, the analysis within the studied group was carried out using a parametric *t*-student-test. The non-parametric Mann–Whitney test was used to compare the intake of nutrients between the study and control groups. Statistical significance was established at a two-tailed value of *p* ≤ 0.05. Post hoc test power analysis was performed using G Power software (Dusseldorf, Germany). The power of tests showed statistical significance above 0.8.

## 3. Results

### The Quantitative Analysis of the Menus

Table 1A–C show the percentage of consumption of ingredients for girls and boys with IBD compared to dietary standards. Both boys and girls in the vast majority of the population had deficient intakes of energy, vitamin D, potassium, calcium, magnesium, iodine, and folate. In addition, boys with IBD showed a low intake of vitamins A, E, B1, and vitamin C, as well as fiber. Other components were consumed at adequate levels by most of the IBD group in our study. It is important to note that none of the nutrients’ single intake values exceeded the upper level (UL) relative to dietary standards [25] and the European Food Safety Authority (EFSA) [26]. 

Table 2 shows the average intake of nutrients such as protein including particular amino acids and carbohydrates, fat, and cholesterol. The nutrient intake in IBD and CG groups of children was analyzed. As for the comparisons between IBD individuals and CG, the results are presented in Table 2.

An energy deficit was statistically significant in both CG and IBD groups. Girls in the study group, in relation to girls in the control group, showed a significantly higher intake of energy (1751. 3 vs. 1558.6 *p* = 0.0224), total protein (71.3 vs. 56.2 *p* = 0.0217), and animal protein (47.8 vs. 34.5 *p* = 0.0183). Thus, statistical significance was shown between girls in the intake of most amino acids (Supplement). Protein intake was at an adequate level in both groups. The ratio of animal protein to plant protein in the girls’ group exceeded 2:1, while in the boys’ group it oscillated around 2:1. Girls in the study group had a significantly higher intake of total carbohydrates (237.3 vs. 196.1 *p* = 0.0442) and assimilable carbohydrates (219.8 vs. 180.5 *p* = 0.7921) compared to girls in the control group. In contrast, boys with IBD showed a higher percentage of dietary carbohydrate intake relative to boys in the control group (55 vs. 48.5). Fat intake in both groups was at an adequate level. Intake of saturated fatty acids was excessive for both groups. Mean dietary carbohydrate intake was at normal levels in both groups. Additionally, in the study group, boys consumed significantly more starch than girls (150.8 vs. 110.7 *p* = 0.0029). 

Saccharose intake did not exceed 10% of the dietary caloric intake in both groups relative to the mean. However, higher saccharose intake was observed in the IBD group relative to the control group. There was also a significantly lower intake of lactose in the IBD group of boys relative to the CG of boys (6.1 vs. 11.6 *p* = 0.0016). 

The mean fiber intake in both IBD and CG groups was below the accepted norm and did not differ significantly between them.

Table 3 shows the intake of vitamins. Significant statistical differences were observed in vitamin B6 (1.9 vs. 1.3 *p* = 0.0199) and niacin (18.9 vs. 12.2 *p* = 0.0047) intake between girls (IBD vs. CG). In contrast, in boys, significant differences were found for riboflavin (1.3 vs. 1.7 *p* = 0.0123) (IBD vs. CG). A statistically significant difference in vitamin C (112.5 vs. 100.7 *p* = 0.0356) intake (girls IBD vs. boys IBD) was observed in the study group. 

None of the genders in both IBD and CG groups achieved the required levels of vitamin D and folate intake. Inadequate intake of vitamin D in the group affected 100% of girls and 96.9% of boys, and in both cases was at critically low levels of 1.4 ± 0.9 µg and 3.1 ± 3.2 µg, respectively. Adequate intake of vitamins such as vitamin A, B1, B2, B3, B6, B12 and C were observed in both the study and control groups. Both boys in the study and control groups showed insufficient intake of vitamin E, which was not observed in the group of girls.

Regarding the mineral components presented in Table 4, the sodium intake was significantly exceeded in both groups, and higher sodium intake was found among boys at a statistically significant level compared to girls (2927.9 vs. 2447 *p* = 0.0367) (IBD vs. IBD). Intake of iron, copper, and manganese met the norms in both groups. Girls with IBD consumed significantly higher amounts of manganese (4 vs. 2.4 *p* = 0.0447), potassium (2588.5 vs. 2054 *p* = 0.0416), and phosphorus (1167.1 vs. 933.4 *p* = 0.0345) compared to girls in the control group. Insufficient intake of potassium, magnesium, and calcium was found in both the control and study groups. Also, boys in the control group consumed significantly more calcium (851.8 vs. 432 *p* = 0.0006) and phosphorus (1357.5 vs. 1024.3 *p* = 0.0431) compared to boys with IBD. The highest deficiency among mineral components was observed in calcium, potassium, and magnesium. Potassium deficiency was higher in the girls’ group, while magnesium deficiency was higher in the boys’ group. Mean zinc intake was insufficient in the boys’ group (IBD). Insufficient intake of iodine, which was not observed in the boys, was observed in both the study and control group of girls. 

## 4. Discussion

Malnutrition is common in children with IBD, especially in patients with CD, both in the exacerbation and in the remission phases of the disease [27]. In patients with UC, malnutrition is relatively less common, although deficiencies may rapidly worsen during exacerbation of the disease [28]. The onset of malnutrition may be influenced by inadequate dietary energy intake, generalized inflammation, and malabsorption [28]. Protein-calorie malnutrition in patients negatively affects not only muscle and respiratory function, but also impairs immune function, including humoral and cellular responses, thus affecting mucosal barrier integrity [29]. 

In this study, we aimed to analyze the differences between the nutritional patterns of children with IBD and healthy controls and to assess the alterations relative to dietary standards for the Poles. Children with IBD have a high risk of growth delay, and an inadequate intake can make this worse. Similarly, Diederen et al. showed lower energy intake in adolescents with IBD relative to healthy controls [30]. In our study, we also were able to evaluate adequate protein intake in both the control and study groups as well as its normal percentage distribution in the diet. However, we observed an increased intake of animal to plant protein at a ratio of 2:1 in the IBD group. Increased protein intake has also been reported in other studies [31,32,33]. In the present study, significantly more animal protein was consumed by IBD girls relative to CG girls. It seems that the high intake of animal protein may be related to the maintenance of dietary recommendations and habits from the exacerbation period [33]. In contrast, a study by Diederen et al. found a higher proportion of plant protein intake relative to animal protein in children’s diets [30]. 

A similar percentage distribution of fat intake was shown in a study by Vagianos et al. [31]. Moreover, the study revealed excess intake with respect to saturated fatty acids (SFA), which concerned as much as 61.9% of girls and 56.3% of boys in the study group. Similarly, a study by Aghdassi et al. involving patients with CD showed an excessive intake of saturated acids [34]. Meanwhile, FFAs were demonstrated to activate pro-inflammatory TLR4 (toll-like receptor) pathways, enhancing the organism’s inflammatory response [35].

In our study, higher carbohydrate intake was observed in the IBD group, especially among girls. This phenomenon also translated into higher assimilable carbohydrate intake in the IBD group relative to CG. The same observations were made in another study [33].

The observed fiber deficiencies indicate that this nutrient is not commonly used in the diets of IBD individuals. However, dietary fiber intake in this study does not differ markedly from the reference standard. A low-fiber diet is often used by patients as a preventive measure. This is explained by the fear of recurrent exacerbation of the disease [36]. Reduced intake of fiber-rich foods was observed in a study by Cohen et al. [37] and in the study by Hartman et al. in which the deficiency of this component was up to 44% [32]. Another study involving children and adolescents with IBD and healthy controls also found similar fiber intake in both groups [36]. Interestingly, there are studies showing a positive effect of fiber intake on lowering the risk of exacerbation of CD, but not UC [38]. 

Regarding fat-soluble vitamins, a drastically low intake of vitamin D was noted in both the study and control groups. Low vitamin D intake is commonly justified by the limited variety of foods in which the vitamin is present in relevant quantities [39]. Inadequate intake of cholecalciferol in the IBD group affected 100% of girls and 96.9% of boys. Vitamin D plays a key role in calcium homeostasis and the regulation of blood pressure and electrolytes in the body [40]. It plays a major role in supporting the immune system [40]. A large number of studies show vitamin D deficiency occurs more frequently among patients with CD than UC [41]. Malabsorption disorders associated with IBD might promote reduced vitamin D absorption through impaired bile salt secretion [42]. It was shown that, in patients with CD who had lower vitamin D levels, the disease was of a more severe course [43]. The deficiency of this vitamin has been associated with higher rates of hospitalization and treatment with steroids [44]. Moreover, studies estimated that as many as 77% of patients with IBD have osteopenia and 12–41% have osteoporosis [41,45]. The risk of osteoporosis and osteopenia among patients with IBD is also related to insufficient intake and absorption of calcium and potassium, lower body mass index (BMI) and insufficient physical activity, low peak bone mass, and certain medications, especially glucocorticosteroids [46,47]. Adequate calcium intake is crucial due to IBD’s association with an increased incidence of osteoporosis, and additionally, during ongoing inflammation, pro-inflammatory cytokines affect the activity of bone cells [42]. Due to impaired vitamin D absorption being associated with the disease, osteomalacia, which is associated with impaired bone mineralization, may also occur [48]. Studies by other researchers have also observed lower dietary calcium intake in patients with IBD [49,50].

Regarding the intake of minerals in this study, there was a significant deficiency of calcium and magnesium consumption in both groups. In addition, a significantly lower intake of this component was shown in IBD boys relative to CG boys. In contrast, mean zinc intake was below the recommended values only in the IBD male group. Inadequate zinc intake maintains inflammation. Adequate levels of this element may reduce the production of pro-inflammatory cytokines and the elevation of mucosal permeability in the course of CD [51]. Magnesium deficiency is also common in patients with IBD, and the deficiency estimates vary between 13–88% [52]. Insufficient intake of this nutrient might be associated with considerable weakness, muscle cramps, and delayed wound healing, especially in CD [53]. We found in both groups a significant excess of sodium intake standards, which has also been observed by other researchers [54]. In a study in mice, high NaCl intake was shown to be associated with exacerbation of colon inflammation, suggesting that excess salt intake may exacerbate the disease phenotype [55]. Furthermore, excess dietary sodium can activate macrophages, causing increased inflammatory activity and stimulating pathogenic TH cells [16]. This high sodium intake was mainly due to the consumption of products like cold cuts, cheese, bread, cornflakes, etc. The habit of adding salt (as deliberate salting) was sporadic in this group. In turn, potassium has been shown to exhibit anti-inflammatory effects [56]. Interestingly, inadequate iron intake was also not shown, contrasting with the reports of other researchers [31,57]. Similarly, a study involving patients with CD found deficiencies in components such as calcium, magnesium, zinc, and potassium and, interestingly, higher sodium intake in the female group [58].

## 5. Conclusions

Proper eating habits are crucial for the overall well-being of an organism. Nutrition is a major factor contributing to the course of inflammatory diseases. Therefore, patients diagnosed with IBD should be treated in an interdisciplinary way in cooperation with a doctor and a dietician. Due to the risk of delayed growth and youth development associated with inadequate nutrition, dietary interventions should be introduced early. Moreover, nutrition education should be emphasized for a healthy pediatric population. We have not studied the influence of nutritional scales on the course of the disease and therefore cannot recommend a recommended intake of components. However, we would like to highlight the need for supplementation of selected components. We suggest considering supplementation in amounts above 50% of the requirement for calcium, magnesium, and potassium, as well as vitamin D and folate. Vitamins B1 and B3 and iron should be considered in the amount of about 20% of the demand. In addition, boys should be supplemented with vitamin E and zinc. 

A major limitation of the study is its relatively small sample size. However, more studies on nutritional assessment in children with IBD are warranted, especially in the context of ethnic diversity. In addition, people on elimination diets and patients with food allergies and severe disease may be at higher risk of deficiencies than our study group. Economic situation was not included due to the reluctance of a significant proportion of patients to answer questions related to social status.

## Figures and Tables

**Table 1:** The percentage of girls and boys in the study group among whom the intake of nutrients deviates from accepted nutritional standards [%].

**A. The percentage of girls and boys with reference to the EAR norm.**						
Constituent		% of girls			% of boys	
	<EAR	norm	>EAR	<EAR	norm	>EAR
Protein (g)	4.8	49	46.2	3.1	15.6	81.3
P (mg)	14.3	33.3	52.4	37.5	15.6	46.9
Mg (mg)	57.1	19.1	23.8	78.1	9.4	12.5
Zn (mg)	9.5	42.9	47.6	34.4	25	40.6
Cu (mg)	9.5	14.3	76.2	15.6	15.6	68.8
Iodine (µg)	61.9	9.5	28.6	62.5	-	37.5
Vitamin A (µg)	14.3	19	66.7	46.9	6.2	46.9
Vitamin B1 (mg)	28.6	23.8	47.6	40.6	15.6	43.8
Vitamin B2 (mg)	4.8	4.7	90.5	31.3	18.7	50
Niacin (mg)	23.8	9.5	66.7	21.9	3.1	75
Vitamin B6 (mg)	-	19	81.0	12.5	12.5	75
Folates (µg)	76.2	4.8	19	87.5	9.4	3.1
Vitamin B12 (µg)	14.3	19	66.7	37.5	18.7	43.8
Vitamin C (mg)	14.3	19	66.7	53.1	6.3	40.6
**B. The percentage of girls and boys with reference to the AI norm.**						
Constituent		% of girls			% of boys	
	<AI	norm	>AI	<AI	norm	>AI
Na (mg)	9.5	14.3	76.2	-	9.4	90.6
K (mg)	61.9	19.1	19	59.4	12.5	28.1
Ca (mg)	85.7	4.8	9.5	93.7	-	6.3
Fe (mg)	19	14.3	66.7	28.1	12.5	59.4
Manganese (mg)	-	14.3	85.7	25	15.6	59.4
Vitamin D (µg)	100	-	-	96.9	3.1	-
Vitamin E (mg)	28.6	38.1	33.3	65.6	12.5	21.9
**C. The percentage of girls and boys with reference to nutritional norms.**						
Constituent		% of girls			% of boys	
	<norm	norm	>norm	<norm	norm	>norm
Energy (kcal)	61.9	33.3	4.8	71.9	21.8	6.3
Protein (%)	4.8	19.1	14.3	2.9	97.1	-
Fat (%)	-	85.7	14.3	5.9	94.1	-
SFA (g)	-	38.1	61.9	-	43.7	56.3
Carbohydrates (%)	4.8	95.2	-	2.9	91.2	5.9
Saccharose (g)	-	61.9	38.1	-	81.2	18.8
Dietary fiber (g)	38.1	19	42.9	62.5	12.5	25

EAR—Estimated average requirement, AI—Adequate intake, SFA—Saturated fatty acids.

**Table 2:** The comparison of mean nutrient consumption (proteins, fats, and carbohydrates) in IBD and CG by gender.

Constituents	Norm	Girls (IBD) Avg ± SD	Girls (CG) Avg ± SD	*p* Value Girls (IBD) vs. Girls (CG)	Boys (IBD) Avg ± SD	Boys (CG) Avg ± SD	*p* Value Boys (IBD) vs. Boys (CG)	*p* Value Girls (IBD) vs. Boys (IBD)	*p* Value Girls (CG) vs. Boys (CG)
Energy (kcal)	2100/2600 *	1751.3 ± 334.6	1558.6 ± 505.3	0.0224	1892.3 ± 590	1943.6 ± 616.9	NS	NS	0.0113
Total proteins	0.84 g/kg (43/45 *g)	71.3 ± 20.1	56.2 ± 21.7	0.0217	1032.6 ± 535.8	85.8 ± 32.5	NS	NS	0.0091
Animal protein (g)	-	47.8 ± 17.4	34.5 ± 17.4	0.0183	72.8 ± 26.2	59.7 ± 29.8	NS	NS	0.0058
Plant protein (g)	-	21.9 ± 6.1	21 ± 9.2	NS	47.1 ± 22.4	24.7 ± 12.1	NS	NS	NS
% energy from protein	10–20	16.4 ± 3.8	15.7 ± 3.7	NS	15.5 ± 3.4	17.9 ± 5.4	NS	NS	NS
Fat (g)	47–82/58–101 *	62.7 ± 17.6	53.3 ± 22.5	NS	62.5 ± 30.4	69.8 ± 28.4	NS	NS	NS
NKT (g)	13.6–16.3/16.8–20.1 *	23.3 ± 10	20.5 ± 11.4	NS	26.1 ± 13.3	28 ± 15.6	NS	NS	0.0452
MUFA (g)	-	23.3 ± 7.3	20.9 ± 8.9	NS	13.8 ± 4.9	26.3 ± 10.5	NS	NS	NS
PUFA (g)	-	10.5 ± 5.8	7.8 ± 4.6	NS	7.8 ± 4.9	10 ± 5	NS	NS	NS
MUFA/ PUFA [%] ^#^	-	12/5.4	12.1/4.6	NS	6.6/3.8	12.1/4.6	NS	NS	NS
LCP (g)	-	0.1 ± 0	0 ± 0.1	NS	0.1 ± 0.4	0.2 ± 0.5	NS	NS	NS
Cholesterol (mg)	-	239.7 ± 132.2	205.4 ± 137.5	NS	317.8 ± 247.9	307.9 ± 216.8	NS	NS	0.0452
% energy from fat	20–35	31.9 ± 7	31.8 ± 7.9	NS	28.2 ± 8.3	31.8 ± 6.1	NS	NS	NS
Total carbohydrates (g)	-	237.3 ± 61.2	196.1 ± 75	0.0442	268.6 ± 79.5	251.4 ± 89.4	NS	NS	0.0312
Assimilable carbohydrates (g)	-	219.8 ± 58.5	180.5 ± 69.6	0.7921	252.7 ± 76.3	233.9 ± 83.8	NS	NS	0.0173
Dietary fiber (g)	19	17.6 ± 7	15.7 ± 7.5	NS	15.6 ± 6.3	17.5 ± 9.7	NS	NS	NS
Saccharose (g)	52.5/65 *	48.3 ± 32.5	29.5 ± 18.1	NS	52.8 ± 42.7	44.8 ± 25	NS	NS	0.0376
Lactose (g)	-	6.2 ± 5.6	7.2 ± 7.2	NS	6.1 ± 6.2	11.6 ± 10	0.0016	NS	0.0312
Starch (g)	-	110.7 ± 30.4	106.8 ± 43.5	NS	150.8 ± 51.9	132 ± 58.5	NS	0.0029	NS
% energy from carbohydrates	45–65	50.6 ± 8.1	50.4 ± 8	NS	55 ± 9.3	48.5 ± 7.8	NS	NS	NS

IBD—Inflammatory bowel diseases; CG—control group; * girls/boys; ^#^ % of intake in relation to total energy content of the diet; NS—non statistically significant.

**Table 3:** A comparison of vitamin consumption.

Constituents	EAR Norm	Girls (IBD) Avg ± SD	Girls (CG) Avg ± SD	*p* Value Girls (IBD) vs. Girls (CG)	Boys (IBD) Avg ± SD	Boys (CG) Avg ± SD	*p* Value Boys (IBD) vs. Boys (CG)	*p* Value Girls (IBD) vs. Boys (IBD)	*p* Value Girls (CG) vs. Boys (CG)
Vitamin A (µg)	490/630 *	971.3 ± 923.8	887.9 ± 588.1	NS	1068.2 ± 1059.8	943.9 ± 494.8	NS	NS	NS
Retinol (µg)	-	287.8 ± 207.2	306.4 ± 177.6	NS	463.6 ± 696.3	347.4 ± 231.2	NS	NS	NS
Beta carotene (µg)	-	4009.1 ± 5568.1	3482.2 ± 3442.7	NS	3579.6 ± 4209.9	3592.8 ± 2689.6	NS	NS	NS
Vitamin E (mg)	8 **/10 *	8.7 ± 3.4	13.5 ± 33.8	NS	6.9 ± 3.9	9.3 ± 5.1	NS	NS	NS
Thiamine (mg)	0.9/1 *	1.1 ± 0.5	0.9 ± 0.6	NS	1.1 ± 0.6	1.3 ± 0.5	NS	NS	0.0452
Riboflavin (mg)	0.9/1.1 *	0.9	1.4 ± 0.5	1.3 ± 0.7	1.3 ± 0.6	1.7 ± 0.7	0.0123	NS	0.0173
Niacin (mg)	11/12 *	18.9 ± 12.4	12.2 ± 9.7	0.0047	17.3 ± 9.7	19.7 ± 11.3	NS	NS	NS
Vitamin B6 (mg)	1/1.1 *	1.9 ± 1	1.3 ± 0.7	0.0199	1.7 ± 0.8	1.9 ± 0.7	NS	NS	0.0211
Vitamin C (mg)	55/65 *	112.5 ± 118.6	100.7 ± 92.8	NS	71.5 ± 71.8	99.6 ± 98.7	NS	0.0356	NS
Vitamin B12 (µg)	2	2.5 ± 0.8	2.5 ± 1.3	NS	2.6 ± 1.8	3.6 ± 1.6	NS	NS	0.0096
Vitamin D (µg)	15 **	1.4 ± 0.9	1.6 ± 1.6	NS	2.1 ± 2.2	3.1 ± 3.2	NS	NS	NS
Folates (µg)	330	245.5 ± 135.5	220.2 ± 92.7	NS	202.5 ± 86	244.3 ± 107.2	NS	NS	NS

IBD—Inflammatory bowel diseases; CG—control group; * girls/boys; ** AI norm; NS—non statistically significant.

**Table 4:** A comparison of mineral consumption levels.

Minerals	EAR Norm	Girls (IBD) Avg ± SD	Girls (CG) Avg ± SD	*p* Value Girls (IBD) vs. Girls (CG)	Boys (IBD) Avg ± SD	Boys (CG) Avg ± SD	*p* Value Boys (IBD) vs. Boys (CG)	*p* Value Girls (IBD) vs. Boys (IBD)	*p* Value Girls (CG) vs. Boys (CG)
Sodium (mg)	1500 **	2447 ± 1089.7	2258.6 ± 1119.4	NS	2927.9 ± 1375.5	3156.8 ± 1393.5	NS	0.0367	NS
Potassium (mg)	3000 **	2588.5 ± 992.9	2054 ± 952	0.0416	2494.7 ± 983.9	2913.8 ± 1053.5	NS	NS	NS
Calcium (mg)	1100 **	592.8 ± 284.3	619.8 ± 356.3	NS	432 ± 267.4	851.8 ± 516	0.0006	NS	0.0376
Phosphorus (mg)	1050	1167.1 ± 316.6	933.4 ± 377.6	0.0345	1024.3 ± 410.2	1357.5 ± 451.7	0.0431	NS	0.014
Magnesium (mg)	300/340 *	261.2 ± 96.9	205.9 ± 92.3	NS	232.2 ± 108.5	293.1 ± 117.5	NS	NS	NS
Iron (mg)	8 **	8 *	10.2 ± 3.9	8.3 ± 5.1	9.4 ± 3.7	11.4 ± 5.3	NS	NS	NS
Zinc (mg)	7.3/8.5 *	8.5 ± 2.5	6.5 ± 2.5	0.0427	8.1 ± 3.3	9.3 ± 3.4	NS	NS	0.0257
Copper (mg)	0.7	1 ± 0.3	0.9 ± 0.4	NS	0.9 ± 0.4	1.1 ± 0.5	NS	NS	NS
Manganese (mg)	1.6 **/2.2	4 ± 2.2	2.4 ± 1.6	0.0447	3 ± 2	3.5 ± 3	NS	NS	NS
Iodine (µg)	95	81.2 ± 43.8	79.6 ± 56.5	NS	98.4 ± 67.1	106.2 ± 62.9	NS	NS	NS

IBD—Inflammatory bowel diseases; CG—control group; * girls/boys; ** AI norm; NS—non statistically significant.

## Data Availability

Data available on request.
